# Exposure to ergot alkaloids during gestation reduces fetal growth in sheep

**DOI:** 10.3389/fchem.2014.00068

**Published:** 2014-08-21

**Authors:** Susan K. Duckett, John G. Andrae, Scott L. Pratt

**Affiliations:** ^1^Animal and Veterinary Sciences Department, Clemson UniversityClemson, SC, USA; ^2^School of Agricultural Forest and Environmental Sciences, Clemson UniversityClemson, SC, USA

**Keywords:** sheep, ergot alkaloids, fetal growth, muscle development

## Abstract

Tall fescue [*Lolium arundinaceum* (Schreb.) Darbysh; *Schedonorus phoenix* (Scop.) Holub] is the primary cool season perennial grass in the eastern U.S. Most tall fescue contains an endophyte (*Neotyphodium coenophialum*), which produces ergot alkaloids that cause vasoconstriction and could restrict blood flow to the fetus in pregnant animals. The objective of this study was to examine fetal growth during maternal exposure to ergot alkaloids during gestation. Pregnant ewes (*n* = 16) were randomly assigned to one of two dietary treatments: (1) endophyte-infected (*N. coenophialum*) tall fescue seed (E+; 0.8 ug of ergovaline /g diet DM) and (2) endophyte-free tall fescue seed (E−; 0.0 ug of ergovaline/g diet DM). Birth weight of lambs was reduced by 37% for E+ compared to E−. Organ and muscle weights were also lighter for E+ than E−. Exposure to ergot alkaloids *in utero* reduces fetal growth and muscle development.

## Introduction

Tall fescue [*Lolium arundinaceum* (Schreb.) Darbysh; *Schedonorus phoenix* (Scop.) Holub] is the primary cool season perennial grass utilized in the eastern U.S. occupying more than 14 million ha (Stuedemann and Hoveland, 1988). The majority of tall fescue contains an endophyte (*Neotyphodium coenophialum*), which produces ergot alkaloids (i.e., ergovaline, ergovalinine, lysergic acid etc.). The endophyte is beneficial to the plant and improves establishment, persistence, and drought tolerance (Stuedemann and Hoveland, 1988); however, ingestion of the ergot alkaloids by grazing livestock results in fescue toxicosis which reduces animal growth (Hoveland, 1993) and reproductive performance (Peters et al., 1992). Ergot alkaloids contain a tetracyclic ergoline ring and are structurally similar to biogenic amines, serotonin, dopamine, norepinephrine, and epinephrine (Berde, 1980; Weber, 1980; Strickland et al., 2011). Ergot alkaloids bind to receptors for the biogenic amines and elicit decreased serum prolactin concentrations and vasoconstriction (Klotz et al., 2006, 2012; Aiken et al., 2009). Dyer (1993) found that ergovaline induced contraction of bovine uterine and umbilical cord arteries via 5HT_2A_ serotonergic receptors, which could restrict blood flow to the fetus. Gestating ewes in the Southeastern US would generally be exposed to endophyte-infected tall fescue throughout the gestation period. Little research exists on how ergot alkaloid exposure in gestating ewes impacts fetal growth and development. The objective of this study was to assess how exposure to ergot alkaloids during gestation (d 35 to parturition) of ewes altered fetal growth and development.

## Materials and methods

All animal experimental procedures were reviewed and approved by the Clemson University Institutional Animal Care and Use Committee (AUP-2011-053).

Southdown ewes (*n* = 20; BW = 70 kg; BCS = 4) were mated to a single ram that was fitted with a marking harness. Ewes were checked twice daily and crayon marks from the ram's harness were denoted to estimate breeding date. Ewes were confirmed pregnant via transrectal ultrasonography on d 35 of gestation. Ewes confirmed pregnant (*n* = 16) were randomly assigned to one of two dietary treatments: (1) endophyte-infected (*N. coenophialum*) tall fescue seed (E+; 0.8 μg of ergovaline + ergovalinine/g diet DM) and (2) endophyte-free tall fescue seed (E−; 0.0 μg of ergovaline + ergovalinine/g diet DM). Endophyte-infected and endophyte-free tall fescue seed (E+ *cv*. Defiance, and E− *cv*. Fawn, turf-type tall fescue seed, Seed Research of Oregon, Tangent, OR) was first analyzed for ergovaline and ergovalinine levels according to Aiken et al. (2009) and then diets formulated to provide the targeted levels of ergovaline/ergovalinine in the diet. Fescue seed was delivered daily in a total mixed ration (Table 1) formulated to meet NRC requirements for pregnant ewes from d 35 to parturition.

**Table 1:** **Composition of the total mixed ration containing endophyte-infected tall fescue seed fed to the ewes during gestation**.

**Ingredient**	**% of ration, DM**
Tall fescue seed	38.5
Cottonseed hulls	15.4
Molasses	8.6
Corn grain, cracked	18.9
Soybean hulls	11.4
Limestone	0.2
Soybean meal	2.8
**NUTRIENT COMPOSITION, DM BASIS**	
Crude protein	11%
TDN	60%

Blood samples were collected from the ewes via jugular venipuncture into tubes on d 30, 50, and 130 of gestation. Samples were allowed to clot for 30 min at room temperature and then at 4°C overnight. Serum was obtained by centrifuging at 1000 × *g* for 15 min at 4°C and stored frozen at −20°C. Prolactin (PRL) concentrations were measured using RIA according to the procedures of Bernard et al. (1993).

At parturition, a male lamb (E+ = 8; E− = 8) was removed from each ewe carrying twins. If two male lambs were born to the same ewe, the firstborn male lamb was removed from the dam. Male lambs were given a fixed amount of artificial colostrum (Lamb's Choice Total, The Saskatoon Colostrum Co., 3 oz. reconstituted dried bovine colostrum) and harvested within 12 h of birth. The attending veterinarian euthanized lambs with an overdose of pentobarbital. Live weight was collected for each lamb and then the lamb was exsanguinated. The hide, head, feet, and tail were removed and weight of the carcass obtained. Weights were collected on all organs and total digestive tract. From the left side of each carcass, individual muscles [longissimus thoracis (LT), gluteus medius, semimembranosus, semitendinosus, biceps femoris, and quadriceps femoris] were collected and weighed. Samples of the longissimus and semitendinosus muscles were immersed in optimal cutting temperature solution, frozen in liquid nitrogen, and stored at −80°C. for subsequent fiber typing. Adipose depots (subcutaneous fat, kidney fat, mesenteric fat) were also collected and weighed. No appreciable subcutaneous fat depots were present in any of the lambs. From the right side of each carcass, all muscle and fat were removed, weighed and ground for total body proximate composition.

### Proximate composition

For proximate analysis, total muscle and fat samples from the right side of each lamb carcass were chopped (Blixer®3 Series D, Robot Coupe Inc., Ridgeland, MS) to reduce particle size and subset removed for determination of moisture content. The remaining samples were frozen at −20°C, lyophilized (VirTis, SP Scientific, Warminster, PA), ground (Blixer®3), and stored at −20°C. Duplicate samples were analyzed for nitrogen content by the combustion method using a Leco FP-2000 N analyzer (Leco Corp., St. Joseph, MI) and multiplied by 6.25 to determine CP content. Moisture content was determined by weight loss after drying at 100°C for 24 h. Total ash content was determined by ashing at 600°C for 8 h. Total fat content was determined in duplicate using Ankom XT-15 Extractor (Ankom Technology, Macedon, NY) and hexane as solvent.

### Fatty acid composition

Freeze dried total muscle and fat samples from the right side of each lamb carcass were transmethylated according to the method of Park and Goins (1994). Fatty acid methyl esters (FAME) were analyzed using an Agilent 6850 (Agilent, San Fernando, CA) gas chromatograph equipped with an Agilent 7673A (Hewlett-Packard, San Fernando, CA) automatic sampler. Separations were accomplished using a 100-m SP2560 (Supelco, Bellefonte, PA) capillary column (0.25 mm i.d. and 0.20 μm film thickness). Column oven temperature increased from 150 to 160°C at 1°C per min, from 160 to 167°C at 0.2°C per min, from 167 to 225°C at 1.5°C per min, and then held at 225°C for 16 min. The injector and detector were maintained at 250°C. Sample injection volume was 1 μL. Hydrogen was the carrier gas at a flow rate of 1 mL per min. Samples were run twice with a split ratio of 100:1 for trans C18:1 and long-chain fatty acids, and again at split ratio of 10:1 for conjugated linoleic acid (CLA) and omega-3 fatty acids. Individual fatty acids were identified by comparison of retention times with standards (Sigma, St. Louis, MO; Supelco, Bellefonte, PA; Matreya, Pleasant Gap, PA). Fatty acids were quantified by incorporating an internal standard, methyl tricosanoic (C23:0) acid, into each sample during methylation and expressed as a weight percentage of total fatty acids.

### Immunofluorescence image analysis

Longissimus and semitendinosus samples were immersed in optimal cutting temperature solution, frozen in liquid nitrogen, and stored at −80°C. Muscle samples were cryosectioned and fiber typed using antibodies for myosin heavy chain (MHC)-fast (AbCam, My-32) and MHC-slow (Hybridoma Bank, BA-F8). The number and cross-sectional area of primary and secondary myofibers were counted on 10 different sections for each lamb, and a ratio of secondary to primary myofibers is reported. The cross-sectional area was measured using IMT iSolution Lite (version 9.4, IMT i-Solutions Inc., Vancouver, BC, Canada).

### Statistical analyses

Prolactin data were analyzed in a completely randomized design using MIXED procedure of SAS (SAS Inst. Inc., Cary, NC) with treatment, time, and two-way interaction in the model. Gestation length data was also measured using the MIXED procedure with treatment in the model. Ewe was the experimental unit for both analyses. For all lamb data, data were analyzed in a completely randomized design using MIXED procedure with treatment in the model and lamb as experimental unit. Least square means were generated and separated using the PDIFF option of SAS. Significance was determined at (*P* < 0.05).

## Results and discussion

**Figure 1:**
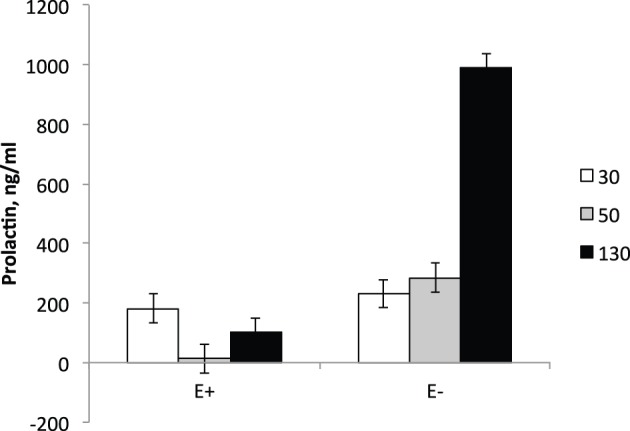
**Serum prolactin levels in ewes at d 30, 50, and 130 of gestation**. Feeding of tall fescue seed was initiated on d 35 and continued through parturition. Treatment × time interaction was significant (*P* < 0.0001).

The interaction between day and treatment was significant (*P* < 0.001) for serum PRL levels (Figure 1). On d 30 of gestation (5 d prior to the initiation of dietary treatments), serum PRL levels did not differ between E+ and E− ewes. At d 50, serum PRL levels in E+ ewes decreased (*P* < 0.01) from pre-treatment levels (d 30) and were lower (*P* < 0.01) than E− levels. In E− ewes, serum PRL levels at d 50 were similar to the values at pre-treatment (d 30) and higher (*P* < 0.01) than E+ ewe values. At d 130, serum PRL levels increased (*P* < 0.05) in both E+ and E− ewes compared to d 50 levels; however, PRL levels were higher (*P* < 0.01) for E− than E+. The reduction in serum PRL concentration with exposure to ergot alkaloids via grazing endophyte-infected tall fescue pastures or consumption of endophyte-infected tall fescue seed is a classical response observed in sheep (Elsasser and Bolt, 1987; Emile et al., 2000; Parish et al., 2003), cattle (Emile et al., 2000; Watson et al., 2004; Koontz et al., 2012; Stowe et al., 2013), and horses (McCann et al., 1992). It has been documented in multiple species that as parturition approaches maternal serum PRL concentration increases (Chamley et al., 1973; Bryant and Chamley, 1976; Forsyth, 1986) and this increase is hypothesized to be important for maternal lipid metabolism, mammary growth, and milk production and secretion (Hooley et al., 1978; Banchero et al., 2006; Mabjeesh et al., 2013). The levels of PRL reported here for the E− group are consistent with previous reports; however, the drastically lower levels observed at d 130 for E+ could indicate post-partum issues with ewe metabolism and mammary growth, which would negatively impact postnatal lamb growth.

**Figure 2:**
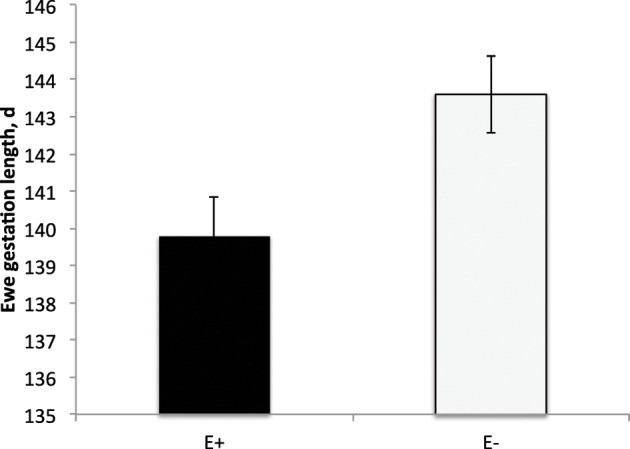
**Gestation length (d) of ewes fed tall fescue seed containing endophyte (E+) vs. endophyte-free (E−)**. Treatment was significant (*P* = 0.02).

**Figure 3:**
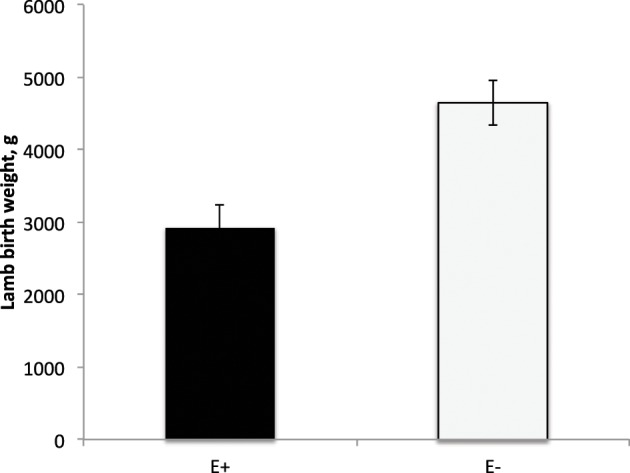
**Lamb birth weight from ewes fed tall fescue seed with endophytes (E+) vs. endophyte-free (E−) during gestation**. Treatment was significant (*P* = 0.001).

E+ ewes had approximately 4 d shorter (*P* < 0.05) gestation length than E− controls (Figure 2). Similarly, others have reported shorter gestation lengths in ewes with placental insufficiency (Chen et al., 2010) and cows that were nutrient restricted from d 32–83 of gestation (Long et al., 2010). In contrast, horses grazing endophyte-infected tall fescue during gestation have increased gestation lengths (Putnam et al., 1991). Lamb birth weight was reduced (*P* < 0.01) by 37% for E+ compared to E− lambs (Figure 3). Watson et al. (2004) observed a 15% reduction in calf birth weight from cows grazing toxic vs. non-toxic fescue during gestation. These reductions in fetal growth with ergot alkaloid feeding are similar to those reported for high ambient temperature exposure throughout pregnancy, which produces the most severe intrauterine growth restriction (IUGR; Bell et al., 1987, 1989; Thureen et al., 1992; Anthony et al., 2003; Arroyo et al., 2006). In sheep, umbilical blood flow increases throughout pregnancy in order to keep pace with fetal growth during the last half of gestation (Reynolds et al., 1986; Reynolds and Ferrell, 1987; Molina et al., 1991). Fetal growth restriction is highly correlated with reduced uteroplacental growth and development (Reynolds and Redmer, 1995, 2001). Experimental conditions like overnutrition, nutrient restriction, hyperthermia, or high altitude that retard fetal growth also reduce uterine and umbilical blood flows (Reynolds et al., 2006). Because adequate blood flow is essential for normal fetal growth, conditions that restrict fetal and placental growth are associated with reduced rates of placental blood flow and nutrient uptakes by the fetus (Reynolds and Redmer, 1995). Since ergot alkaloids cause vasoconstriction in uterine and umbilical blood flow (Dyer, 1993), these effects would induce fetal growth restriction similar to maternal hyperthermia or nutrient deprivation.

Organ weights (heart, lung, kidneys, spleen, thymus, liver, and pancreas) were also smaller (*P* < 0.05) for E+ than E− (Table 2) except for the pancreas (*P* = 0.52). Total muscle weight from the right side of each carcass was lighter (*P* = 0.0093) for E+ than E−. Individual muscle weights for LT, semitendinosus, semimembranosus, biceps femoris, quadriceps femoris, and gluteus medius were heavier (*P* < 0.05) for E− than E+. Kidney fat amounts were lower (*P* < 0.05) for E+ than E−. Thymus and spleen mass tended (*P* < 0.10) to be smaller for E+ than E− even when adjusted for body or carcass weight. All other organs and muscle weights did not differ (*P* > 0.05) when expressed on a weight basis (Table 3). Total viscera weight (weight of the esophagus, rumen, intestines excluding organs) tended to be greater (*P* < 0.10) for E+ than E− when expressed on a body weight or hot carcass weight basis.

**Table 2:** **Effect of feeding tall fescue seed with endophyte (E+) vs. endophyte-free (E−) to ewes during gestation (d 35 to parturition) on lamb organ, muscle and adipose tissue weights**.

	**E+**	**E−**	**SEM**	***P*-Level**
*n*	8	8		
**ORGANS, g**				
Heart	22.3	35.1	2.58	0.0035
Lungs	65.4	112.3	8.76	0.0019
Kidneys	16.7	24.0	1.64	0.0067
Spleen	4.7	9.2	1.03	0.0081
Thymus	4.9	11.2	2.00	0.04
Liver	71.6	112.5	10.2	0.017
Pancreas	0.84	1.3	0.050	0.52
Total viscera	234.4	311.0	29.0	0.082
**MUSCLES, g**				
Longissimus	37.4	63.2	5.06	0.0029
Gluteus medius	11.5	17.6	1.73	0.02
Semitendinosus	8.5	13.6	1.64	0.04
Semimembranosus	24.3	43.4	4.32	0.007
Quadriceps femoris	24.9	41.8	3.42	0.007
Biceps femoris	17.2	29.7	3.30	0.02
Total muscle	313.5	510.7	46.27	0.0093
**ADIPOSE, g**				
Mesenteric fat	4.6	5.2	0.58	0.51
Kidney fat	12.5	19.8	2.28	0.04

**Table 3:** **Effect of feeding tall fescue seed with endophyte (E+) vs. endophyte-free (E−) to ewes during gestation (d 35 to parturition) on lamb organ, muscle and adipose tissue weights as a percentage of body weight**.

	**E+**	**E−**	**SEM**	**P-Level**
*n*	8	8		
**ORGANS, %**				
Heart	0.76	0.76	0.04	0.96
Lungs	2.2	2.4	0.15	0.42
Kidneys	0.57	0.52	0.03	0.32
Spleen	0.15	0.19	0.02	0.07
Thymus	0.14	0.23	0.04	0.10
Liver	2.3	2.4	0.10	0.40
Pancreas	0.24	0.027	0.009	0.81
Total viscera	8.0	6.7	0.45	0.06
**MUSCLES, %**				
Longissimus	2.6	2.7	0.13	0.57
Gluteus medius	0.78	0.75	0.04	0.64
Semitendinosus	0.59	0.57	0.07	0.89
Semimembranosus	1.6	1.8	0.14	0.28
Quadriceps femoris	1.7	1.8	0.06	0.23
Biceps femoris	1.1	1.2	0.11	0.48
Total muscle	21.2	21.7	0.95	0.72
**ADIPOSE, %**				
Mesenteric fat	0.14	0.11	0.01	0.08
Kidney fat	0.42	0.42	0.05	0.98

The proximate and fatty acid composition of the total muscle mass from the right side of each lamb carcass is shown in Table 4. Moisture content was higher (*P* < 0.01) and crude protein content was lower (*P* = 0.05) in total muscle from E+ than E−. Total lipid and ash content of the muscle did not differ between treatments. Stearic (C18:0) acid concentrations of the total muscle tended to be lower (*P* = 0.10) for E+ than E−. Arachidonic (C20:4) and eicosapentaenoic (C20:5) acid concentrations were higher (*P* < 0.05) in total muscle of E+ than E−. Other fatty acid concentrations were not altered by dietary treatment. Total *n*-6 polyunsaturated fatty acid (PUFA) and the ratio of *n*-6 to *n*-3 PUFA were higher (*P* < 0.05) in the muscle of E+ than E−. Total fatty acid content of the muscle did not differ, which indicates that PUFA fatty acid accumulation in muscle was greater with E+ exposure. Realini et al. (2005) reported that finishing steers on endophyte-infected vs. endophyte-free tall fescue increased stearic acid and lowered monounsaturated fatty acid concentrations with no change in PUFA. Ailhaud et al. (2008) found that increased levels of *n*-6 PUFA and a high ratio of *n*-6 to *n*-3 PUFA during fetal development in rats stimulated adipogenesis to alter hypertrophy and hyperplasia of adipocytes during postnatal growth. These alterations in fatty acid composition at birth could impact adipogenesis and subsequent adipose tissue deposition.

**Table 4:** **Proximate composition of total muscle mass from one side of each lamb carcass from ewes fed tall fescue seed with endophyte (E+) vs. endophyte-free (E−) during gestation (d 35 to parturition)**.

	**E+**	**E−**	**SEM**	***P*-Level**
Moisture, %	79.05	78.64	0.09	0.01
Crude protein, %	17.09	18.74	0.69	0.05
Total Lipid, %	2.44	2.50	0.20	0.83
Ash, %	2.12	2.17	0.77	0.78
**FATTY ACIDS, %**				
C14:0	0.90	1.01	0.07	0.28
C16:0	19.67	20.81	0.84	0.35
C16:1 cis-9	2.14	2.03	0.14	0.59
C17:0	0.36	0.39	0.03	0.33
C18:0	13.13	14.44	0.52	0.10
C18:1 cis-9	49.95	49.54	0.72	0.69
C18:1 cis-11	3.03	2.99	0.12	0.83
C18:2 cis-9,12	0.65	0.51	0.09	0.26
C18:3 cis-9,12,15	0.28	0.26	0.05	0.77
C20:4 cis-5,8,11,14	1.89	0.61	0.32	0.01
C20:5 cis-5,8,11,14,17	0.40	0.18	0.07	0.04
C22:5 cis-7,10,13,16,19	0.31	0.50	0.16	0.42
C22:6 cis-4,7,10,13,16,19	0.26	0.20	0.05	0.44
Saturated	33.70	36.26	1.17	0.14
Monounsaturated	52.09	51.57	0.77	0.64
Polyunsaturated, *n*-6	2.54	1.12	0.37	0.02
Polyunsaturated, *n*-3	1.25	1.16	0.22	0.76
Ratio of *n*-6:*n*-3	1.96	1.07	0.20	0.01
Total fatty acids, g/100g LT	1.76	1.81	0.19	0.85

**Figure 4:**
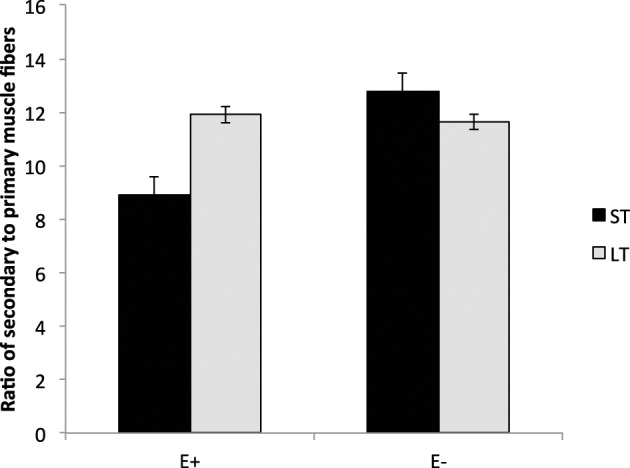
**Secondary to primary muscle fiber ratio in the longissimus thoracis (LT) and semitendinosus (ST) muscles in lambs from dams fed tall fescue seed containing endophyte (E+) vs. endophyte-free (E−) during gestation**. Treatment was significant (*P* = 0.002) for ST muscles but non-significant for LT (*P* = 0.57).

**Figure 5:**
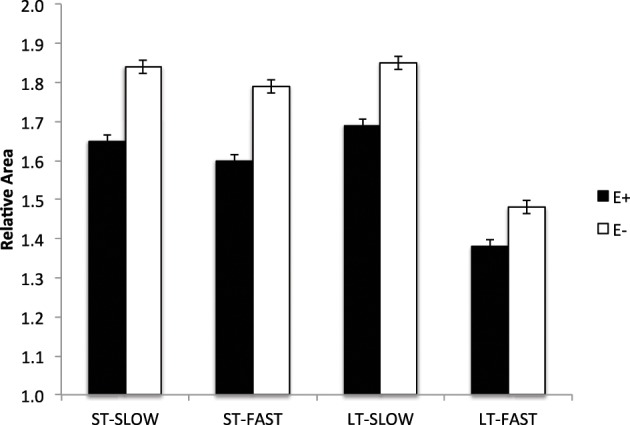
**Cross sectional area of muscle fibers (slow- and fast-MHC) in the longissimus thoracis (LT) and semitendinosus (ST) muscles in lambs from dams fed tall fescue seed containing endophyte (E+) vs. endophyte-free (E−) during gestation**. Treatment was significant (*P* < 0.001) for both LT and ST muscles.

Lambs exposed to ergot alkaloids *in utero* had a lower (*P* < 0.05) secondary to primary muscle fiber ratio in the semitendinosus muscle compared to E− (Figure 4). The ratio of secondary to primary muscle fiber did not differ in the LT. Early prenatal muscle fiber growth is due to hyperplasia of muscle fibers and fiber number is set before birth. Research indicates that muscle fiber hyperplasia is complete by about 70 d of gestation in the pig (Swatland, 1973), 180 d in the cow (Albrecht et al., 2013), and 105 d in the sheep (Du et al., 2010). Intrauterine growth restriction of the fetus during the second trimester of gestation reduces the formation of secondary muscle fibers. The ratio of secondary to primary muscle fibers is reduced with intrauterine crowding in pigs (i.e., runt pig, Aberle, 1984; Pardo et al., 2013) and maternal under-nutrition from d 28 to 78 in sheep (Zhu et al., 2004). Cross-sectional area was also reduced (*P* < 0.05) in slow and fast-MHC myofibers of the LT and ST muscles in E+ compared to E− (Figure 5). Because postnatal muscle growth is predominately through hypertrophy of existing muscle fibers, a reduction in secondary fiber number also impacts postnatal muscle growth. Pigs that are runts at birth have less total carcass muscle mass and altered adipose tissue cellularity when finished to slaughter weights (Powell and Aberle, 1981). Underwood et al. (2010) found that mid to late nutrient restriction of gestating cows altered growth, adipose, and meat tenderness in the offspring. Long et al. (2012) also reported changes in adipocyte size and carcass parameters in beef offspring from cows with early to mid-gestation undernutrition. Thus, ingestion of ergot alkaloids by ewes during critical time periods of gestation alters fetal muscle growth and development that may have lasting impact on postnatal muscle growth, carcass composition, and palatability throughout the offspring's lifetime.

These results show that fetal growth is restricted in ewes fed endophyte-infected tall fescue seed to simulate fescue toxicosis syndrome during gestation (d 35 to parturition). This reduction in lamb birth weight with ergot alkaloid exposure is similar to lambs exposed *in utero* to high ambient temperatures, which is the most severe IUGR. Exposure *in utero* to ergot alkaloids altered skeletal muscle formation by reducing the ratio of secondary to primary myofibers, myofiber hypertrophy *in utero*, and protein content of muscles. Due to the number of ruminant animals that graze endophyte-infected tall fescue during gestation, additional research is needed to determine mechanisms by which ergot alkaloids reduce fetal growth and the critical time periods of exposure in order to mitigate its effects on fetal growth.

### Conflict of interest statement

The authors declare that the research was conducted in the absence of any commercial or financial relationships that could be construed as a potential conflict of interest.
